# Acute Fatty Liver of Pregnancy Presenting With Multiorgan Failure: A Case Report Highlighting Diagnostic Differentiation From Hemolysis, Elevated Liver Enzymes, and Low Platelets (HELLP) Syndrome and Implications for Early Intervention

**DOI:** 10.7759/cureus.109795

**Published:** 2026-05-28

**Authors:** Loveleen K Johal, Danish Sohail, Nabeeha Azhar, Rabia Azhar, Ali Ahmed

**Affiliations:** 1 Internal Medicine, St. George's University, St. George's, GRD; 2 Internal Medicine, Punjab Employees Social Security Institution, Lahore, PAK; 3 Internal Medicine, Faisalabad Medical University, Faisalabad, PAK; 4 Internal Medicine, Jinnah Hospital, Lahore, PAK; 5 Internal Medicine, Avicenna Medical College, Lahore, PAK; 6 Internal Medicine, Lahore Medical and Dental College, Lahore, PAK; 7 Internal Medicine, Services Hospital, Lahore, PAK

**Keywords:** acute fatty liver of pregnancy, acute kidney injury, aflp, disseminated intravascular coagulation, hellp syndrome, hepatic encephalopathy, hypoglycaemia, multiorgan failure, pregnancy-associated liver disease, swansea criteria

## Abstract

Acute fatty liver of pregnancy (AFLP) is a rare but potentially life-threatening complication of late gestation that often presents with nonspecific features and significant overlap with other obstetric conditions, posing a diagnostic challenge. We report a case of a 28-year-old multigravida at 34 weeks of gestation who presented with progressive jaundice, encephalopathy, and multiorgan dysfunction, including disseminated intravascular coagulation (DIC) and acute kidney injury (AKI) requiring renal replacement therapy. Laboratory evaluation revealed severe hypoglycemia, hyperammonemia, marked transaminase elevation, and profound coagulopathy, supporting the diagnosis of AFLP based on the Swansea criteria. The clinical picture was initially confounded by overlapping features with hemolysis, elevated liver enzymes, and low platelets (HELLP) syndrome; however, key biochemical markers facilitated diagnostic clarification. The patient underwent prompt multidisciplinary management, including metabolic stabilization, correction of coagulopathy, and emergency cesarean delivery, followed by intensive care support. Significant clinical and biochemical improvement was observed following delivery, with progressive recovery of hepatic and renal function. This case highlights the importance of early recognition, differentiation from mimicking conditions, and timely intervention in AFLP, particularly in severe presentations with multiorgan involvement. It further emphasizes the role of hypoglycemia and metabolic dysfunction as critical diagnostic indicators and supports the principle that prompt delivery remains the definitive treatment in preventing maternal morbidity and mortality.

## Introduction

Acute fatty liver of pregnancy (AFLP) is an uncommon but potentially life-threatening complication of late gestation, typically occurring in the third trimester and associated with significant maternal and perinatal morbidity. The condition is characterized by microvesicular fatty infiltration of hepatocytes resulting from impaired mitochondrial β-oxidation of fatty acids, often linked to fetal deficiencies in enzymes such as long-chain 3-hydroxyacyl-CoA dehydrogenase (LCHAD) [1]. Clinically, AFLP presents with nonspecific symptoms including nausea, vomiting, abdominal pain, and malaise, which frequently overlap with other pregnancy-related conditions such as hemolysis, elevated liver enzymes, and low platelets (HELLP) syndrome, severe pre-eclampsia, and acute viral hepatitis. This overlap, combined with the potential for rapid progression to hepatic dysfunction, coagulopathy, and multiorgan involvement, poses a significant diagnostic challenge. Laboratory findings such as hypoglycemia, hyperbilirubinemia, elevated transaminases, and deranged coagulation parameters provide important diagnostic clues, while the Swansea criteria offer a practical framework for clinical diagnosis in the absence of histological confirmation [2]. Early recognition and prompt delivery remain the cornerstone of management and are critical in improving maternal outcomes [3].

The objective of this case report is to present a severe and diagnostically complex presentation of AFLP in a multigravida at 34 weeks of gestation, complicated by disseminated intravascular coagulation (DIC), acute kidney injury (AKI) requiring renal replacement therapy, and hepatic encephalopathy. This report aims to highlight the challenges in differentiating AFLP from other hypertensive and hepatic disorders of pregnancy, particularly HELLP syndrome, and to underscore the diagnostic value of key biochemical features such as hypoglycemia and hyperammonemia. In addition, the case seeks to illustrate the importance of a coordinated multidisciplinary approach in the management of advanced disease and to contribute to the existing literature by providing a clinically instructive perspective on the recognition, diagnostic reasoning, and management of severe AFLP with multiorgan involvement.

## Case presentation

A 28-year-old multigravida (G3P2) of South Asian descent at 34 weeks of gestation presented to the emergency department with a five-day history of progressive nausea, recurrent vomiting, epigastric and right upper quadrant pain, profound malaise, and rapidly worsening jaundice. Over the preceding 48 hours, she developed confusion, reduced urine output, and generalized edema. There was no history of seizures, vaginal bleeding, or preceding febrile illness. She reported dark urine but denied pruritus. Antenatal care had been irregular, and there was no documented history of hypertension or proteinuria in the current pregnancy. Her previous two pregnancies had been uncomplicated. She had no history of liver disease, alcohol use, or hepatotoxic drug exposure. On presentation, she was tachycardic (108 beats per minute), hypertensive (148/92 mmHg), tachypneic (24 breaths per minute), and mildly febrile (37.8°C), with oxygen saturation of 94% on room air.

On examination, she appeared acutely ill and deeply icteric, with moderate impairment of consciousness reflected by a Glasgow Coma Scale score of 12/15. Abdominal examination revealed a gravid uterus consistent with gestational age, with tenderness in the epigastric and right upper quadrant regions and a palpable liver edge 3 cm below the costal margin. Bilateral pitting edema was present. Respiratory examination demonstrated reduced air entry at the right lung base with dullness to percussion. Fetal heart rate was 148 beats per minute and regular.

**Figure 1:**
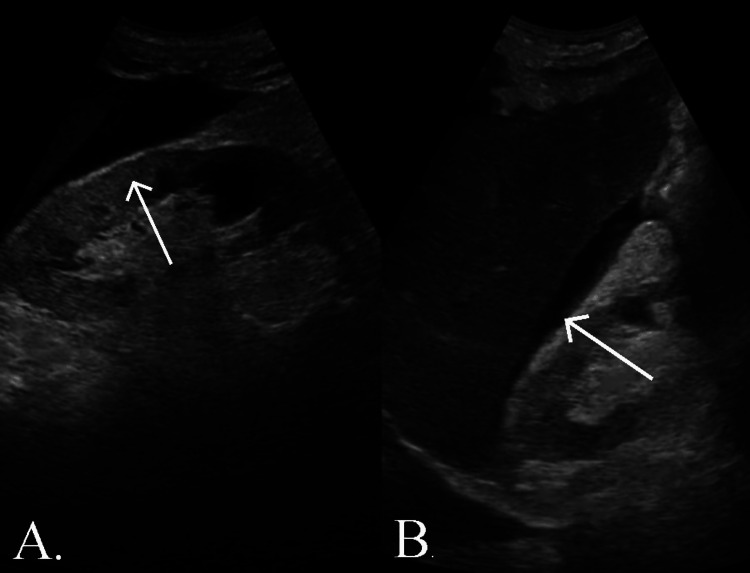
Abdominal ultrasonography demonstrating complications associated with acute fatty liver of pregnancy: (A) free intraperitoneal fluid consistent with ascites (arrow); (B) enlarged liver consistent with hepatomegaly (arrow).

Urgent obstetric and abdominal ultrasonography demonstrated a single live fetus with normal biometry, a normal amniotic fluid index, and no evidence of placental abruption. Maternal imaging revealed a diffusely echogenic liver suggestive of steatosis, homogeneous hepatomegaly, marked ascites, and bilateral pleural effusions (Figure 1). Laboratory investigations (Table 1) showed severe hepatic dysfunction with marked transaminase elevation and hyperbilirubinemia, together with metabolic derangement, coagulopathy, and acute renal impairment, consistent with multiorgan involvement.

**Table 1:** Baseline laboratory findings demonstrating hepatic dysfunction, metabolic derangement, coagulopathy, and renal impairment in acute fatty liver of pregnancy. ALT: alanine aminotransferase; AST: aspartate aminotransferase; INR: international normalized ratio; WBC: white blood cell; RBC: red blood cell; HPF: high-power field; FEU: fibrinogen equivalent units

Parameter	Result	Reference range
Hemoglobin	7.2 g/dL	11.5-16.5
Platelets	68 × 10⁹/L	150-400
WBC count	18.2 × 10⁹/L	4-11
ALT	620 U/L	<40
AST	890 U/L	<40
Total bilirubin	8.4 mg/dL	0.3-1.2
INR	1.8	0.9-1.3
Fibrinogen	0.9 g/L	>2.0
Serum glucose	42 mg/dL	70-140
Serum ammonia	98 μmol/L	11-35
Creatinine	3.8 mg/dL	0.5-1.0
Uric acid	9.4 mg/dL	2.6-6.0
Lactate	4.2 mmol/L	0.5-2.0
Sodium	128 mmol/L	135-145
Potassium	5.6 mmol/L	3.5-5.0
D-dimer	9.8 μg/mL FEU	<0.5
Urinalysis: protein	2+	Negative
Urinalysis: bilirubin	Positive (2+)	Negative
Urinalysis: casts	Granular casts present	None
Urinalysis: RBCs	0-2/HPF	0-2/HPF
Urinalysis: WBCs	2-4/HPF	0-5/HPF

Urinalysis demonstrated bilirubinuria, proteinuria, and granular casts, supporting concurrent renal involvement. Viral hepatitis serology was negative, and thyroid function testing was within normal limits. The clinical and biochemical profile fulfilled nine Swansea criteria, including vomiting, abdominal pain, encephalopathy, hyperbilirubinemia, hypoglycemia, elevated transaminases, hyperammonemia, renal impairment, and coagulopathy, confirming the diagnosis of AFLP. Although HELLP syndrome was considered because of hypertension, thrombocytopenia, and elevated liver enzymes, the presence of severe hypoglycemia, hyperammonemia, and profound coagulopathy supported AFLP as the primary diagnosis. A multidisciplinary team approach was initiated immediately.

Initial management focused on stabilization and correction of life-threatening abnormalities. Severe hypoglycemia was treated with an intravenous dextrose bolus followed by continuous infusion with close monitoring. Coagulopathy was corrected with fresh frozen plasma, cryoprecipitate, and platelet transfusions to achieve hemostatic targets suitable for surgical intervention. Intravenous vitamin K was administered, and antihypertensive therapy with labetalol infusion was initiated. Hepatic encephalopathy was managed with lactulose and rifaximin.

Following initial stabilization, an emergency lower-segment cesarean section was performed under general anesthesia. Intraoperative findings included moderate ascites. A live male neonate was delivered at 34 weeks of gestation with Apgar scores of 6 and 8 at one and five minutes, respectively, and was admitted to the neonatal intensive care unit. Cord blood was collected for the evaluation of fatty acid oxidation disorders.

Postoperatively, the patient required intensive care support. She developed persistent oliguria with worsening renal function, necessitating initiation of continuous renal replacement therapy due to hemodynamic instability, fluid overload, and hyperkalemia. This was continued for five days, followed by intermittent hemodialysis sessions as renal parameters improved. Hepatic encephalopathy resolved progressively with medical therapy, and nutritional support was initiated via enteral feeding. Serial monitoring demonstrated gradual normalization of metabolic and hepatic parameters. Significant clinical and biochemical improvement was observed following delivery. By postoperative day four, the patient had regained full consciousness. By day seven, laboratory parameters, given in Table 2, showed marked recovery, as outlined below.

**Table 2:** Postoperative laboratory parameters showing recovery of hepatic, renal, and metabolic function following delivery and supportive management. ALT: alanine aminotransferase; AST: aspartate aminotransferase; INR: international normalized ratio

Parameter	Result	Reference range
Serum glucose	88 mg/dL	70-140
Serum ammonia	28 μmol/L	11-35
ALT	180 U/L	<40
AST	210 U/L	<40
Total bilirubin	2.1 mg/dL	0.3-1.2
INR	1.2	0.9-1.3
Fibrinogen	2.4 g/L	>2.0
Platelets	148 × 10⁹/L	150-400
Creatinine	1.4 mg/dL	0.5-1.0
Sodium	136 mmol/L	135-145
Potassium	4.2 mmol/L	3.5-5.0
Hemoglobin	9.6 g/dL	11.5-16.5

The patient demonstrated sustained clinical recovery and was transferred from intensive care to the obstetric ward on postoperative day eight. At discharge, she remained hemodynamically stable with improving hepatic and renal function. Follow-up planning included continued monitoring of biochemical parameters, evaluation of the neonate for long-chain fatty acid oxidation disorders, and genetic counseling for future pregnancies.

## Discussion

This case illustrates a severe and clinically instructive presentation of AFLP, characterized by simultaneous hepatic, renal, coagulation, and neurological involvement. Rather than representing an isolated hepatic disorder, AFLP is more appropriately conceptualized as a systemic metabolic disorder arising from impaired mitochondrial fatty acid oxidation. The coexistence of DIC, AKI requiring renal replacement therapy, hepatic encephalopathy, and electrolyte disturbances reflects a cascade of interdependent organ dysfunction driven by hepatic synthetic failure and accumulation of toxic metabolites [2,4]. Such presentations, although uncommon, represent the severe end of the disease spectrum and underscore the importance of early recognition before progression to multiorgan compromise.

A central challenge in this case was the differentiation of AFLP from other pregnancy-related conditions with overlapping clinical and biochemical features, particularly HELLP syndrome. Both conditions may present with elevated transaminases, thrombocytopenia, and hypertension, creating diagnostic ambiguity in the third trimester. This case highlights the importance of identifying distinguishing biochemical markers. Severe hypoglycemia, reflecting impaired hepatic gluconeogenesis, is characteristic of AFLP and typically absent in HELLP syndrome. Similarly, marked hyperammonemia and early encephalopathy indicate profound hepatic metabolic dysfunction, while the degree of coagulopathy, particularly hypofibrinogenemia and prolonged coagulation parameters out of proportion to thrombocytopenia, further supported AFLP [5]. Viral hepatitis serology was negative, and the absence of severe hypertension with only limited proteinuria further favored AFLP over severe pre-eclampsia and related mimicking conditions. These distinctions are clinically important, as delayed recognition may contribute to inappropriate management and adverse outcomes.

The Swansea criteria provided a practical and reliable diagnostic framework in the absence of histological confirmation, with nine criteria fulfilled in this patient, including vomiting, abdominal pain, encephalopathy, hyperbilirubinemia, hypoglycemia, elevated transaminases, hyperammonemia, renal impairment, and coagulopathy [2,6]. Liver biopsy was appropriately deferred because of the high bleeding risk in the setting of severe coagulopathy, consistent with contemporary practice where diagnosis is primarily clinical. The development of DIC represented a critical marker of disease severity, reflecting profound hepatic synthetic dysfunction. In this context, targeted hemostatic resuscitation, including correction of hypofibrinogenemia and thrombocytopenia, was essential before operative intervention to reduce hemorrhagic risk [7].

Renal dysfunction in AFLP is multifactorial and reflects the close interplay between hepatic and renal physiology, involving hypoperfusion, systemic inflammation, and direct nephrotoxicity from fatty acid metabolites. Following stabilization and emergency delivery, the patient required intensive care support and continuous renal replacement therapy because of persistent oliguria, fluid overload, and hyperkalemia before progressive clinical recovery was observed. The requirement for continuous renal replacement therapy underscores the severity of renal involvement and highlights the importance of early nephrology input, particularly in hemodynamically unstable patients where continuous modalities offer greater physiological stability [8]. From a mechanistic perspective, the association with fetal LCHAD deficiency provides important insight into disease pathogenesis, with accumulation of fatty acid metabolites contributing to maternal hepatic injury and supporting consideration of neonatal metabolic evaluation [9].

The clinical course further supports that delivery remains the definitive intervention in AFLP, as removal of the fetoplacental unit interrupts the pathological process and allows reversal of progressive organ dysfunction. The rapid biochemical and clinical improvement observed following delivery reinforces this pathophysiological model and emphasizes that intervention should not be unnecessarily delayed in pursuit of complete stabilization. Instead, management should focus on achieving conditions that permit safe delivery, particularly correction of coagulopathy, within a coordinated multidisciplinary framework. This case highlights the importance of early diagnostic differentiation, recognition of AFLP as a systemic disorder, and timely collaborative management in improving maternal outcomes [10].

## Conclusions

This case underscores AFLP as a time-critical, systemic obstetric emergency that may present with diagnostic ambiguity and rapidly progress to multiorgan failure if not promptly recognized. It highlights the importance of maintaining a high index of suspicion in third-trimester patients with hepatic dysfunction, particularly when key features such as hypoglycemia, hyperammonemia, and disproportionate coagulopathy are present, which favor AFLP over mimicking conditions such as HELLP syndrome. This case further supports the importance of early application of clinical criteria, rapid multidisciplinary coordination, and timely delivery following targeted stabilization in improving maternal outcomes and reversing progressive organ dysfunction. The primary take-home message is that in suspected AFLP, prompt diagnostic recognition and timely delivery, rather than prolonged diagnostic delay or excessive preoperative optimization, remain central principles of management.
